# Comparison of the Seventh and Eighth Edition of American Joint Committee on Cancer (AJCC) Staging for Selected and Nonselected Oropharyngeal Squamous Cell Carcinomas

**DOI:** 10.1093/oncolo/oyab001

**Published:** 2022-01-28

**Authors:** Pooja Vijayvargiya, Sumita Trivedi, Manali Rupji, Haocan Song, Yuan Liu, Renjian Jiang, Azeem S Kaka, Georgia Z Chen, William Stokes, Conor Steuer, Dong M Shin, Jonathan J Beitler, Mihir R Patel, Ashley Aiken, Nabil F Saba

**Affiliations:** 1 Department of Medicine, Emory University School of Medicine, Atlanta, GA, USA; 2 Department of Biostatistics and Bioinformatics Shared Resource, Winship Cancer Institute, Emory University, Atlanta, GA, USA; 3 Department of Otolaryngology and Head and Neck Surgery, Winship Cancer Institute, Emory University, Atlanta, GA, USA; 4 Department of Hematology and Medical Oncology, Winship Cancer Institute, Emory University, Atlanta, GA, USA; 5 Department of Radiation Oncology, Winship Cancer Institute, Emory University, Atlanta, GA, USA; 6 Department of Radiology and Imaging Sciences, Emory University, Atlanta, GA, USA

**Keywords:** oropharyngeal squamous cell carcinoma, AJCC, human papilloma virus, SEER, staging, TNM, overall survival, disease-specific survival

## Abstract

**Objectives:**

The eighth edition American Joint Committee on Cancer (AJCC) Staging incorporates significant changes to the seventh edition in the staging of oropharyngeal squamous cell carcinomas (OPSCC). An important change was the inclusion of OPSCC associated with the human papilloma virus (HPV). Our goal is to compare the performance of both staging systems for patients with HPV-selected and unselected clinical characteristics for OPSCC.

**Methods:**

Using the Surveillance, Epidemiology, and End Results (SEER) database, 2004-2016, we identified patients with likely HPV-associated OPSCC based on surrogate markers (white males aged <65 years old with squamous cell carcinomas of the tonsil and base of tongue), excluding those who underwent surgery. We re-classified these patients using seventh and eighth edition staging for HPV-selected OPSCC and compared the prediction performance of both staging editions for overall survival (OS) and disease-specific survival (DSS). We performed the same analysis for clinically unselected patients with OPSCC.

**Results:**

Our analysis included 9554 patients with a median follow-up of 67 months. Comparing the eighth versus seventh edition for our HPV-selected cohort, clinical staging changed for 92.3% of patients and 10-year OS was 62.2%, 61.2%, 35.3%, and 15.5% for Stage I, II, III, and IV, versus 52.9%, 59.2%, 61.6%, 55.1%, 38.3%, and 15.5% for stage I, II, III, IVA, IVB, and IVC, respectively. A similar pattern was observed for 10-year DSS. The concordance statistics for our HPV-selected cohort were improved for both AJCC 7 (0.6260) and AJCC 8 (0.6846) compared with the unselected cohort, 0.5860 and 0.6457 for AJCC 7 and 8, respectively.

**Conclusion:**

The overall performance of discrimination improved from AJCC 7 to AJCC 8 for both clinically selected and unselected patients, but more notably for our HPV-selected cohort. Despite the lack of statistically significant differentiation between Stages I and II in AJCC 8 in either groups, markedly improved discrimination was observed between Stages I/II, III, and IV in the HPV-selected cohort.

## Teaser

The 8th edition of the American Joint Committee on Cancer (AJCC) Staging Manual incorporates significant changes to the 7th edition in the staging of oropharyngeal squamous cell carcinomas (OPSCC). An important change was the inclusion of OPSCC associated with human papilloma virus (HPV). This article compares the performance of both staging systems for patients with HPV-selected and -unselected clinical characteristics for OPSCC.

**Implications for Practice**

This article addresses the impact the new AJCC eighth edition for OPSCC staging has compared with the prior seventh edition. Our findings support evidence that the eighth edition provides better prognostication and discrimination between stages compared with AJCC 7 for both selected HPV-associated and unselected OPSCC patients. In comparison to prior publications using single-institution studies, ours is the only study to date using the SEER database to compare the performance of AJCC 7 and 8 for OPSCC. It is of interest that our findings applied regardless of attempting to clinically select patients for HPV relatedness.

## Introduction

The Centers for Disease Control (CDC) has reported a significant change in the incidence and proportion of common human papilloma virus (HPV)-associated cancers within the last 2 decades. The incidence of HPV-associated oropharyngeal squamous cell carcinoma (OPSCC) increased at an estimated rate of 2.7% per year in men and 0.8% per year in women from 1999 to 2015.^1^ While cervical carcinoma was the most common HPV-associated malignancy, as of 2015, oropharyngeal SCC has now become the most prevalent HPV-associated malignancy.^1^ During this time, the incidence of OPSCC has increased despite a notable improvement in survival.^2^ This discrepancy is likely a reflection of the emergence of HPV-associated OPSCC as the predominant subtype of OPSCC.^3,4^ This trend was also paralleled by changes in tobacco use and sexual practices.^3,4^

This change in behavior noted in HPV-associated OPSCC is reflected in recent changes in AJCC staging for this disease. While the seventh edition of the AJCC Staging Manual was agnostic to HPV status,^5^ AJCC 8 incorporated HPV-associated OPSCC as a separate entity in terms of staging and prognosis. AJCC 8 was largely based on the multicenter cohort study by the International Collaboration on Oropharyngeal cancer Network for Staging (ICON-S), which showed similar overall survival (OS) for different nodal (N1-N2b) and tumor (T4a-T4b) categories, ultimately simplifying this tumor nodes metastases (TNM) classification system.^6^ Consequently, there are major TNM classification differences between the seventh and eighth editions of the AJCC Staging Manual, most notably in the T and N criteria.

Within the T classification, HPV-associated carcinoma in situ was disregarded.^7^ T0 was removed from the non-HPV-associated disease given the strong correlation of unknown primary (T0) with HPV-associated disease.^7^ Lastly, T4a and T4b were combined in HPV-associated OPSCC given their similarity in outcome.^7^

N classification was partitioned into clinical and pathologic categories. For clinical N criteria, HPV-associated disease has similar prognostic implications for one or more ipsilateral lymph node (LN) <6 cm in size (previously subdivided into N1 and N2 in the seventh edition based on number and size). These were combined to cN1 in the eighth edition. Since the presence of contralateral or bilateral LNs is correlated with worse outcomes, bilaterality was upstaged to cN2. Finally, LN >6 cm, which foretold the worst prognostic findings, remained in the cN3 category. In contrast, pathologic N criteria in AJCC 8 considered the number of pathologically positive LNs, regardless of size or laterality, with ≤ 4 LNs conferring pN1 classification and >5 LNs a pN2 classification.^7^ It is important to consider the diagnostic differences between clinical and pathologic N categories. While clinical N classification can apply to all patients at diagnosis regardless of therapeutic intervention, pathologic N classification exclusively applies to patients who have undergone surgery. Regardless of the differences in nodal size or laterality, these 2 N classification systems are not designed to be compared since they apply to different clinical settings.

We conducted a retrospective cohort study to stage patients identified in the SEER database with likely HPV-associated OPSCC using AJCC 7 and 8 criteria to compare their discrimination of OS and disease-specific survival (DSS).

## Methods

### Study Design

The Surveillance, Epidemiology, and End Results (SEER) 2004-2016 database collects cancer data from population-based cancer registries across the United States to derive information on survival and incidence. We utilized SEER to identify patients with suspected HPV-associated OPSCC and compared AJCC 7 and 8 staging in terms of their discrimination of outcome, namely OS for the 2 cohorts. To assess this, we completed a retrospective cohort study staging HPV-selected and non-selected OPSCC subjects based on TNM classification of the seventh and eighth AJCC editions, respectively. To clarify, we will refer to the suspected HPV-associated group as “selected” and the general OPSCC population in the SEER database as “non-selected.” Details on group staging can be found in Supplemental Tables 1 and 2.^8^ For the staging not available in the database (eg, the eighth staging or seventh edition before 2010), we constructed them based on individual T, N, and M categories in seventh (2010+) or sixth edition, utilizing the most recent edition T, N, M classification was available in.

### HPV Status

Given that HPV status was not available in our full patient subset, surrogate markers (white race; male sex; age less than 65 years; anatomic site specifically of the tonsil and base of tongue) were used as clinical surrogates for HPV-associated OPSCC. While this methodology cannot replace direct HPV testing, these demographic markers were carefully chosen to create a specific subset of likely HPV-positive disease. White males have the highest incidence of HPV-positive OPSCC, documented in multiple prior epidemiologic assessments.^2,9–13^ Mahal assessed over 12 000 patients from the SEER database with SCCHN diagnosed from 2013 to 2014 (similar to our median year of diagnosis 2010), describing white race (5.47; 95% confidence interval [CI] 5.33-5.61) and male sex (8.00; 95% CI, 7.78-8.20) with the highest incidence of HPV-positive OPSCC.^9^ Younger age <65 has also been more commonly seen in HPV-positive OPSCC as noted in Mahal’s population-based epidemiologic study with the age distribution peaking at 60-64 years old for all patients (13.55, 95% CI 12.81-14.28).^9^ Other studies have echoed these findings.^12–15^ On the other hand, the incidence of HPV-positive disease in OPSCC is increasing in the elderly population. This is notably represented in Thompson’s study in which the mean age of diagnosis increased from 55.2 years (2002-2010) to 58.5 (2011-2016).^16^ However, since our study’s mean date of diagnosis was ~2010, we decided to utilize the association with a younger patient population and implement an age restriction < 65 years old. Another HPV criteria were to target those with squamous cell disease at the base of the tongue and tonsils, as many prior studies have localized HPV-positive OPSCC to these locations.^2,13,14,17–19^ As Zamani noted in a population-based study with more than 2000 patients from 2000 to 2017, HPV-positive OPSCC were more often located in the base of tongue and tonsils than in the uvula, soft palate, or pharyngeal walls (HPV-positive 93% vs HPV-negative 58%).^14^ In addition, we also explored the performance of the 2 staging systems in a completely unselected group of OPSCC as a secondary confirmatory analysis.

### Patient Selection with Biometrics

We searched the SEER database from 2004 to 2016 with limitations on sex, race, age, and disease location, as detailed above. Disease location was limited to the tonsil and base of the tongue. Patients were excluded for unknown/immeasurable T and/or N classification and were excluded if they had undergone surgery as a treatment modality. This was largely to focus our analysis on clinical rather than pathologic TNM classification. In addition to TNM categories, we collected risk factors (age and smoking status) as well as the type of treatment (chemotherapy and radiation). We considered excluding smokers to obtain a more refined subset of HPV-driven disease; however, we did not want to omit the portion of concomitant smokers and HPV-positive patients who represent an important trend toward worse outcomes. Studies have shown that smoking is a negative prognostic factor in HPV-positive patients^20^ and more specifically has been shown to increase death and recurrence rates of OPSCC in HPV-positive patients.^21^ Ang et al. detailed this relationship by purporting that tobacco use changes the biological behavior of HPV-positive OPSCC and can subsequently decrease responsiveness to the standard of care therapy.^22^

### Statistical Analysis

Summary statistics and demographics were represented as mean ± standard deviation (SD) and sample frequency and proportion. The seventh and eighth editions AJCC staging was constructed from individual clinical T, N, and M criteria based on their definition. OS was defined as months from the date of diagnosis to death for any cause or to the last date of follow-up. DSS was defined similarly to OS except that deaths specific to head and neck cancer were counted as events unless death was linked to other causes or patients were lost follow-up.

The comparison of AJCC 7 and 8 on OS was evaluated by the Cox proportional hazard model along with Kaplan–Meier method. The discrimination performance was measured and compared by concordance statistics (C-statistics) in the Cox model. Results were generated using SAS Macros.^23^ Prediction and c-index calculations were performed in R packages *survival*, *survcomp*^2^, *cph*^3^. The significance level was set at *P* < .05.

## Results

### Patient Demographics

Between 2004 and 2016, 48 655 patients with OPSCC were identified in the SEER database. After including male gender, age < 65 years, and disease location isolated to tonsil and/or base of the tongue and excluding unknown/nonspecified T and/or N criteria, non-Caucasian patients, and those who underwent surgery as a primary treatment, 9554 patients were analyzed (Supplementary Figure 1). Demographics, risk factors for oropharyngeal cancers, and treatment are described in Table 1.

**Table 1.** Demographics, rate of smoking, and treatment within the entire cohort.

	Entire cohort
*N*	9554
Age (mean ± SD)	55.58 ± 6.01
Sex	
Male	9554 (100.0%)
Race	
White	9554 (100.0%)
% Ever Smoker (age 18 ± SAE 2008-2010) (mean ± SD)	39.93 ± 7.20
Radiation	
Yes	8597 (90.0%)
No/Unknown	957 (10.0%)
Chemotherapy	
Yes	8148 (85.3%)
No/Unknown	1406 (14.7%)
Year of diagnosis	
≥2004, ≤2008	2982 (31.2%)
>2008, ≤2011	2597 (27.2%)
>2011, ≤2014	2899 (30.3%)
>2014, ≤2016	1076 (11.3%)

### Major Differences Between Seventh Edition and Eighth Edition Staging

Our cohort of HPV-selected OPSCC patients was restaged from the seventh edition AJCC staging to the eighth edition as indicated. Details of the distribution of staging based on TMN for the seventh and eighth edition AJCC are available in Supplementary Tables 3 and 4. A summary of the distribution of OPSCC staging based on the seventh and eighth editions is shown in Table 2. Applying the eighth edition AJCC criteria, a significant proportion of patients were down-staged from Stage IV to Stage I or II.

**Table 2.** Differences in staging based on the seventh and eighth editions of AJCC for the HPV-selected cohort.

Stage status	Percent of seventh edition staging	Percent of eighth edition staging
Stage I	1.97%	19.22%
Stage II	5.86%	55.75%
Stage III	18.67%	20.93%
Stage IV		
Stage IVA	63.31%	4.10%
Stage IVB	6.08%	
Stage IVC	4.10%	
TOTAL	100%	100%

Please note that in the eighth edition AJCC, there are no subdivisions of Stage IV.^42^

Abbreviations: AJCC, American Joint Committee on Cancer; HPV, human papilloma virus.

Overall, the clinical staging changed for 92.3% of patients, with a majority of patients classified as Stage IVA (63.3%) using AJCC 7 compared with a predominance of Stage II (55.8%) with AJCC 8. In addition, Stage IV in the eighth edition was isolated to patients with metastatic disease, which was previously staged IVC in the seventh edition.

### Survival Analysis

**Figure 1.**
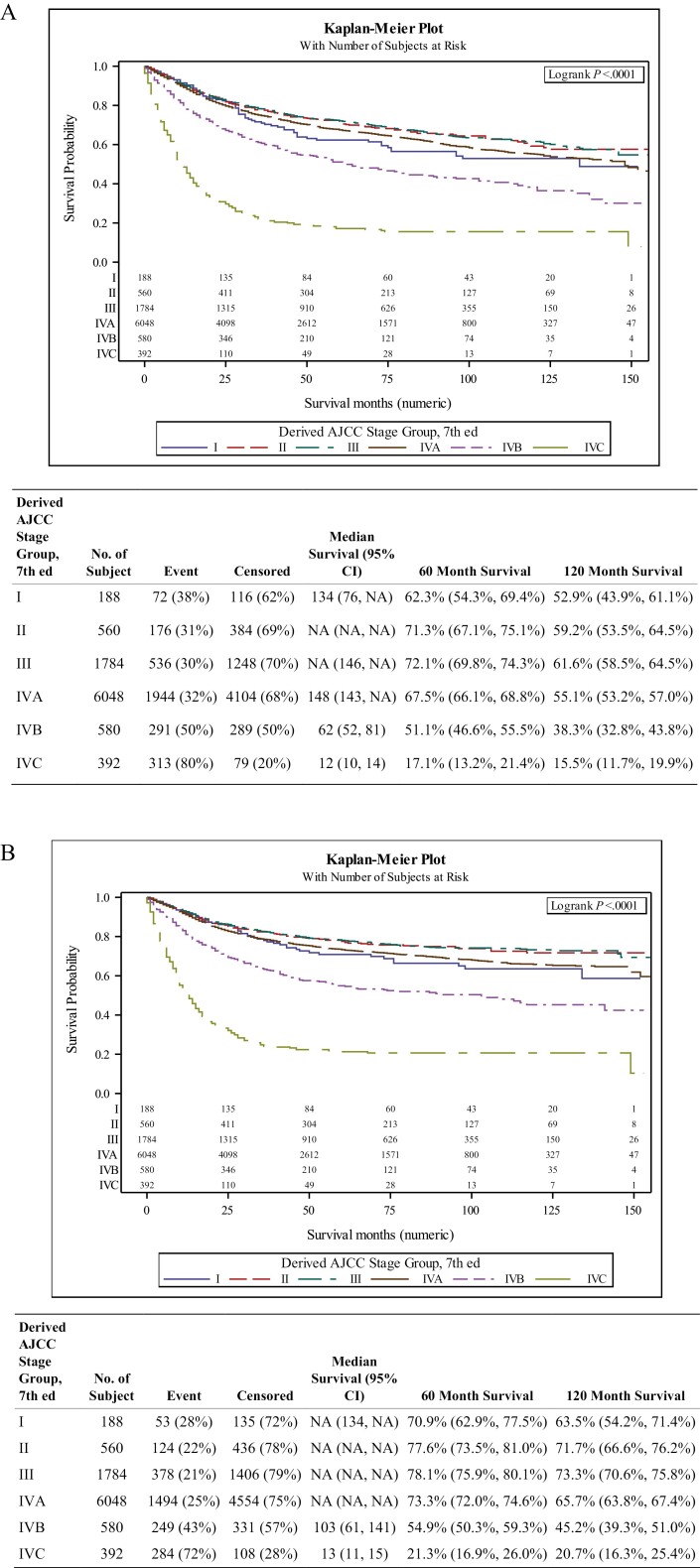
(A) Kaplan–Meier curve of overall survival by different stages based on the seventh edition AJCC criteria for the human papilloma virus (HPV)-selected cohort. Estimated 5- and 10-year survival rate with 95% confidence interval. (**B**) Kaplan–Meier curve of disease-specific survival (DSS) by different stages based on the seventh edition AJCC criteria for the HPV-selected cohort. Estimated 5- and 10-year DSS rate with 95% confidence interval.

Figure 1A shows the Kaplan–Meier curves for patients with Stage I, II, III, and IV disease, based on the AJCC 7 classification, over a 150-month period. The OS rates for Stages II, III, and IVA were largely undifferentiated over the 120-month evaluation as seen in the table within Figure 1A. Stage I generally had worse overall and disease-free survival compared with Stages II, III, and IVA, statistically significant for III but not for II or IVA. Patients with Stage IVB and IVC OPSCC consistently demonstrated a significantly worse outcome as compared with those with Stages I, II, III, and IVA disease. DSS exhibited similar results as OS (Figure 1B).

**Figure 2.**
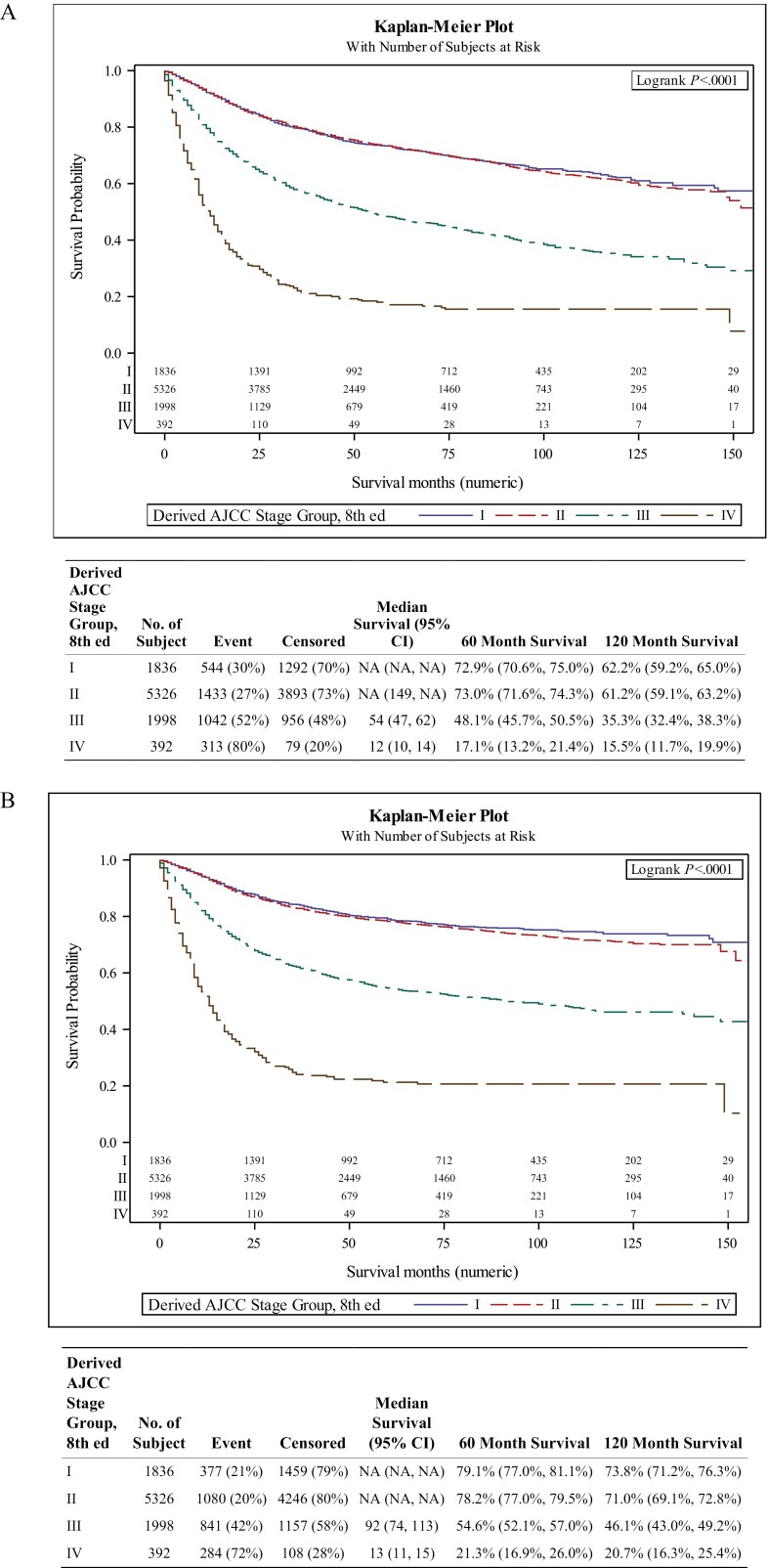
(**A**) Kaplan–Meier curve of overall survival by different stages based on the eighth edition AJCC criteria for the human papilloma virus (HPV)-selected cohort. Estimated 5- and 10-year survival rate with 95% confidence interval. (**B**) Kaplan-Meier curve of disease-specific survival (DSS) by different stages based on the eighth edition AJCC criteria for the HPV-selected cohort. Estimated 5- and 10-year DSS rate with 95% confidence interval.

In contrast to the survival curves based on the seventh edition, staging using AJCC 8 demonstrated a better differentiation of OS over the 10 years between Stage I/II, III, and IV (Figure 2A). There was an early and clearer distinction between stages I/II, III, and IV, with the latter 2 having worse survival at 60 and 120 months, respectively. However, AJCC 8 did not discriminate as clearly for Stages I and II with no statistical significance for OS or DSS (Figure 2B) over the 10-year period.

### Differences in Seventh Versus Eighth Edition Staging on Prognosis

For disease staging, Table 3 shows the hazard ratios for staging with Stage I used as a control for the comparison of the remaining stages. Applying the seventh edition AJCC criteria, only Stages IVB and IVC had statistically significantly higher hazard ratios for survival compared with Stage I. There was no consistently significant difference in OS/DSS between Stages I, II, and IVA. Only Stage III had improved OS/DSS compared with Stage I (*P* = .039; *P* = .038). When employing the eighth edition criteria, Stage I demonstrated marginally improved survival compared with Stage II without statistical significance for OS or DSS. On the other hand, stages III and IV had statistically significantly higher hazard ratios compared with Stage I and were statistically different from each other.

**Table 3.** Univariate hazard ratios for treatment and staging for OS and DSS based on the seventh and eighth edition AJCC for the HPV-selected cohort.

	Exposed	OS HR (95% CI) in survival months	OS HR *P*-value	DSS HR (95% CI) in survival months	DSS HR *P*-value
Radiation	8597/9554	0.28 (0.25-0.30)	**<.001**	0.25 (0.22-0.27)	**<.001**
Chemotherapy	8148/9554	0.51 (0.47-0.56)	**<.001**	0.49 (0.44-0.53)	**<.001**
Seventh edition staging					
** **Stage I	188/9554	--------------------	---------	----------------------	-----------
** **Stage II	560/9554	0.78 (0.59-1.03)	.079	0.75 (0.55-1.04)	.086
** **Stage III	1784/9554	0.77 (0.60-0.99)	**.039**	0.74 (0.55-0.98)	**.038**
** **Stage IVA	6048/9554	0.91 (0.72-1.15)	.426	0.93 (0.71-1.23)	.612
** **Stage IVB	580/9554	1.56 (1.21-2.02)	**<.001**	1.78 (1.32-2.40)	**<.001**
** **Stage IVC	392/9554	4.44 (3.44-5.74)	**<.001**	5.23 (3.90-7.01)	**<.001**
Eighth edition staging					
** **Stage I	1836/9554				
** **Stage II	5326/9554	1.01 (0.91-1.11)	.881	1.07 (0.95-1.20)	.256
** **Stage III	1998/9554	2.33 (2.10-2.59)	**<.001**	2.64 (2.34-2.98)	**<.001**
** **Stage IV	392/9554	6.14 (5.33-7.06)	**<.001**	7.62 (6.53-8.90)	**<.001**

Abbreviations: AJCC, American Joint Committee on Cancer; CI, confidence interval; DSS, disease-specific survival; HPV, human papilloma virus; HR, hazard ratio; OS, overall survival.

### Assessing Prediction Performance

For our HPV-selected cohort, the C-index (95% CI) was statistically lower for seventh edition (0.6260 [95% CI 0.6098-0.6422]) compared with eighth edition (0.6846 [95% CI 0.6704-0.6987]) AJCC staging (*P* < .001). This is in comparison to the survival concordance statistics for the unselected cohort of OPSCC patients (data not shown), where the C-indices were 0.5860 and 0.6457 for the seventh and eighth editions, respectively. This is represented in Table 4.

**Table 4.** Concordance statistics measuring overall discrimination for seventh and eighth editions for both HPV-selected cohort and the entire cohort of OPSCC patients.

STAGE	C-index (95% CI)	SE	*P*-value
Eighth edition: HPV-associated cohort	0.6846(0.6704, 0.6987)	0.0072	<.001
Seventh edition: ­HPV-associated cohort	0.6260(0.6098, 0.6422)	0.0083	<.001
Eighth edition: Entire cohort	0.6457(0.6372, 0.6542)	0.0043	<.001
Seventh edition: Entire cohort	0.5860(0.5767, 0.5953)	0.0048	<.001
Note:			
C-index METHOD	EXPLANATION		
R (Approach3)	based on“concordance.index”function(Rpackage:survcomp)		

*P*-value of 0 is represented as <.001.

Abbreviations: CI, confidence interval; HPV, human papilloma virus.

## Discussion

Overall, our findings demonstrated that staging using the AJCC Staging Manual eighth edition compared with the seventh edition provided superior discrimination with better prediction of survival outcomes regardless of the presence of clinical surrogates for HPV-related OPSCC. The C-index is higher for AJCC 8 compared with AJCC 7 without overlapping confidence intervals with 92.3% of patients restaged, predominantly from Stage IV to Stages I and II for the HPV-selected cohort. This raises the interesting question of whether AJCC 8 would perform better in an overall OPSCC group, regardless of HPV status confirmation.

Survival concordance statistics were calculated for AJCC 7 and AJCC 8 for the unselected cohort of OPSCC patients. The C-indices for the seventh and eighth editions are 0.5860 and 0.6457, respectively. While the C-indices for our HPV-selected population are 0.6260 (95% CI 0.6098-0.6422) and 0.6846 (95% CI 0.6704-0.6987) for AJCC 7 and 8, respectively. This shows that the concordance for AJCC 8 is better than AJCC 7 for all OPSCC patients regardless of HPV status, enforcing the improvements of AJCC 8 in this disease. Our findings are likely related to the fact that HPV-related OPSCC has become the predominant OPSCC disease but also raise the interesting question of AJCC 8 performance in HPV unrelated OPSCC. While our HPV-selected population is by no means inclusive, it likely encompasses a significant subset of highly predictive HPV-positive disease.

Similar findings to ours in HPV-related OPSCC were confirmed in other validation studies,^24–28^ where a large shift from Stage IV to lower stages was observed between AJCC 7 and 8 regardless of treatment. A few of these studies were also unable to prognostically differentiate Stages I and II OPSCC.^25,26^ This could be a feature of the similarly excellent OS and DSS of these early stages of the disease as well as the potential lack of observed discriminatory benefit for early-stage OPSCC. It also raises some questions regarding limitations of the SEER data for these earlier stages as well as our inability to confirm the HPV status in our study.

In contrast, 3 prospective studies were able to differentiate outcomes between Stages I and II disease. Van Gysen et al. demonstrated a statistically significant difference in 5-year OS between Stages I and II in an Australian cohort of 153 patients with p16+ OPSCC treated with weekly cisplatin.^27^ In a second study, Würdemann found statistically significant differences between Stages I and II and utilized combined HPV detection with DNA PCR and p16 in 150 patients who underwent surgery followed by concurrent chemoradiation.^28^ Finally, the third study by Gupta et al. included 218 p16+ OPSCC patients who had surgery as the primary form of treatment and compared both clinical and pathological staging of AJCC 7 and 8.^24^ While they too had overlapping confidence intervals for clinical Stages I and II using AJCC 8, this study could statistically differentiate all pathological stages. These studies are fundamentally different from ours as they directly assessed HPV status and were single-institution trials. Additionally, the studies by Würdemann et al. and Gupta et al. included different patient cohorts who were able to initially proceed with surgery. The latter also separated comparison of clinical and pathological staging and reported a statistical difference in pathological staging only but results similar to ours for clinical staging.

Our results are in contrast to those of a larger population-based study utilizing the National Cancer Database (NCDB), in which Zhan et al. described 3745 surgically treated HPV-positive OPSCC patients with a dramatic downstaging of Stage IV using AJCC 7 to Stage I when applying AJCC 8 with good hazard discrimination of all eighth edition pathological staging.^29^ In our analysis, we excluded patients who had surgery, limiting our assessment of pathological staging.

Some important considerations for the staging of our HPV-selected cohort focus on the weak performance of Stage 1 in AJCC 8 and the poor prognosis incurred with AJCC 8 IV staging. Within our AJCC 8 assessment, Stage I was difficult to distinguish from Stage II. While this has been seen with other similar studies mentioned above, this finding likely represents heterogeneity within Stage I. Yoshida et al. describe this finding in terms of treatment response, namely from the association of chemotherapy and improved survival amongst patients with Stage I HPV-positive OPSCC with lymph node involvement, previously staged III or IVa in AJCC 7.^30^ This outcome, however, was not observed with Stage I patients without lymph node involvement, previously staged I or II in AJCC 7. In fact, this population had worse outcomes with concomitant chemoradiation.^30^ This finding emphasizes the heterogenous collection of OPSCC included in Stage I, despite the different treatment options and that AJCC 8, while prognostically better, should not drive treatment recommendations. This incongruity is paralleled by worse outcomes in Stage I HPV-positive OPSCC patients with radiologic findings of extranodal extension (ENE), seen to have increased risk of distant metastasis and reduced disease-free survival.^31^ This elucidates the need for further efforts to improve staging, as evidenced by poor differentiation of Stage I and II and the known heterogeneity within this subgroup. This raises the question of treatment de-intensification within a subgroup of favorable patients; however, data from the NRG HN002 study showed that not all favorable disease benefits from de-escalation.^32^ An interesting observation that would warrant a further investigation is the stark similarity of 15.5% OS at 120 months for both Stage IVc in the seventh edition and Stage IV in the eighth edition, likely representing not only the poor prognosis of this final stage but also the direct association of AJCC 7’s IVc to AJCC 8’s IV staging. These observed discrepancies reflect the need for continued improvement to our AJCC staging guidelines, especially for clinical staging as those studies assessing pathological staging had more consistent differentiation among the different stages. Despite it being a controversial topic, there is evidence that ENE is a predictor of worse prognosis in HPV-positive OPSCC patients. Bauer et al. showed ENE-positive cases with worse 5-year survival for HPV-positive cases compared with ENE-negative with a hazard of death HR = 1.90; 95% CI: 1.35-2.67.^33^ Other studies have shown a similar correlation between ENE presence and HPV-positivity.^29,34,35^ Whether the inclusion of radiologic ENE in future staging will confer an advantage remains to be seen as conflicting reports exist as to the value of radiologic ENE specifically in HPV-related OPSCC. If this were to be the case, the inclusion of rENE could transition this small subset of Stage I patients with worse survival outcomes into more advanced staging.^36^ This ultimately could improve staging differentiation and impact treatment and subsequent deintensification.

Our findings also raise the question of whether AJCC 8 ought to be examined as a possible staging of choice for OPSCC regardless of HPV status. There are emerging similarities between HPV-positive and HPV-negative disease that may justify more detailed genomic investigation. While HPV status is the most common and robust molecular biomarker for OPSCC, other molecular markers are being highlighted in their role in prognosis and disease behavior. One example is pAMPK activity, which portends a better prognosis for HPV-positive OPSCC, perhaps warranting consideration for de-escalation of therapy.^37^ Another study by Liu et al. focuses on wild-type p53 with low levels supporting a favorable response to radiotherapy.^38^ Further understanding of how these genomic profiles can impact OPSCC outcomes is needed.^39,40^

Limitations of our study include the retrospective nature of data collection utilizing the SEER database with incomplete demographic information, treatment, and HPV status. This study relied partially on demographic factors and HPV-targeted anatomical sites as surrogate markers of HPV status, which was not an ideal proxy for this selected group. While women were excluded to create a more definite HPV-presumed subset, it is important to note women are an affected subgroup that will be important to consider in future studies. Because this was a SEER limitation, we attempted to bypass this constraint by collecting a specific, yet understandably not inclusive, a subset of patients which reflect the changes associated with the new staging guidelines. In addition, our treatment and demographic data were limited by the completeness of variables. SEER notably reports high specificity for treatment data with a high likelihood of receiving treatment if reported. However, overall sensitivities are much lower averaging about 88.0% and 80.1% for radiation and chemotherapy, respectively.^41^ Treatment data are also limited by underlying biases associated with receiving or not receiving chemotherapy and/or radiation, specifically selection bias, patient preference, physician recommendations, comorbidities, disease severity, and so on. Given these limitations, we are unable to compare outcomes based on treatment received or explain the discrepancy in hazard ratios for patients receiving these associated treatments. Furthermore, we cannot base treatment decisions or de-escalation on AJCC 8 as it stands currently, as evidenced by Stage I’s notable heterogeneity.^30^ In regard to smoking, there were 2 time ranges 2004-2007 and 2008-2010 that measured smoking status. We were unable to combine these 2 different variables, so just the status for the 2008-2010 time range was included to avoid any confusion. Due to how these values were calculated, they could not be included in the univariate analysis but are represented in Table 1.

In summary, ours is the only study to date using the SEER database that attempts to compare the performance of AJCC seventh and eighth editions for OPSCC. Our findings support evidence from other analyses that the eighth edition provides better prognostication and discrimination between stages compared with AJCC 7 for patients with a clinical presentation consistent with HPV-associated OPSCC. It is of interest that our findings applied regardless of attempting to clinically select patients for HPV-relatedness.

## Supplementary Material

oyab001_suppl_Supplementary_Figures

oyab001_suppl_Supplementary_Tables

## Data Availability

The data underlying this article will be shared at reasonable request to the corresponding author.
