# Supplementary material for: Comparison of the Seventh and Eighth Edition of American Joint Committee on Cancer (AJCC) Staging for Selected and Nonselected Oropharyngeal Squamous Cell Carcinomas
Source: Oncologist. 2022 Jan 28;27(1):48–56. doi: 10.1093/oncolo/oyab001 (PMC8842371; doi:10.1093/oncolo/oyab001)

The Oncologist
The official journal of the Society for Translational Oncology

Supplemental Figure for:
Comparison of the 7th & 8th ed of American Joint Committee on Cancer (AJCC) staging for selected & non-selected oropharyngeal squamous cell carcinomas
Nabil Saba et al.

Figure S1: Patient identification via the SEER database and reasons for exclusion from the final study cohort. The study included exclusively white male patients between 21 and 64 years old with oropharyngeal squamous cell carcinoma of the tonsil and base of tongue from the SEER database between 2004 and 2016. Patients with unknown/immeasurable T and/or N criteria and those who underwent surgery were excluded.

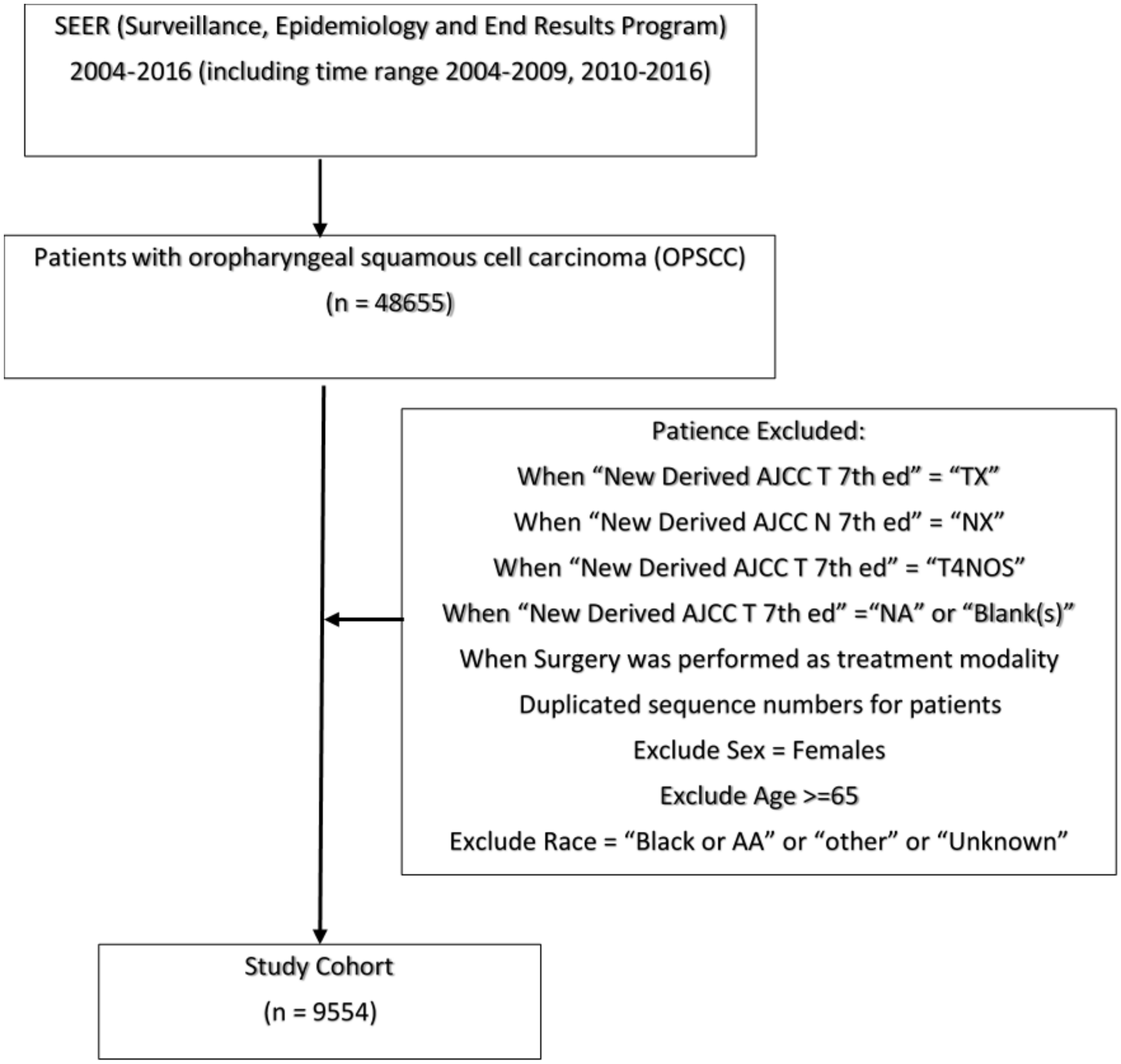

Supplement: oyab001_suppl_Supplementary_Figures [file oyab001_suppl_supplementary_figures.pdf]
