# Supplementary material for: Comparison of the Seventh and Eighth Edition of American Joint Committee on Cancer (AJCC) Staging for Selected and Nonselected Oropharyngeal Squamous Cell Carcinomas
Source: Oncologist. 2022 Jan 28;27(1):48–56. doi: 10.1093/oncolo/oyab001 (PMC8842371; doi:10.1093/oncolo/oyab001)
Supplement: oyab001_suppl_Supplementary_Tables [file oyab001_suppl_supplementary_tables.pdf]

The Oncologist
The official journal of the Society for Translational Oncology

Supplemental Tables for:
Comparison of the 7th & 8th ed of American Joint Committee on Cancer (AJCC) staging for selected & non-selected oropharyngeal squamous cell carcinomas
Nabil Saba et al.

Table S1: AJCC 8 Staging Categories for non HPV-associated OPSCC adapted from updated AJCC 8 guidelines

Table S1: AJCC 8 Stage Categories for non HPV-Associated OPSCC

Stage Group	T Category	N Category	M Category
0	Tis	N0	M0
I	T1	N0	M0
II	T2	N0	M0
III	T3	N0	M0
III	T1, T2, T3	N1	M0
IVA	T4a	N0, N1	M0
IVA	T1, T2, T3, T4a	N2	M0
IVB	Any T	N3	M0
IVB	T4b	Any N	M0
IVC	Any T	Any N	M1

Table S2: AJCC 8 Staging Categories for non HPV-associated OPSCC adapted from updated AJCC 8 guidelines

Table S2: AJCC 8 Stage Categories for HPV-Associated OPSCC

Stage Group	T Category	N Category	M Category
I	T0, T1, or T2	N0 or N1	M0
II	T0, T1, or T2	N2	M0
II	T3	N0, N1, or N2	M0
III	T0, T1, T2, T3, or T4	N3	M0
III	T4	N0, N1, N2, or N3	M0
IV	Any T	Any N	M1

Table S3 – 7th edition AJCC Staging Group after restaging with Supplemental Table 1

7th Stage Group	T Category	N Category	M Category	Frequency	Percent
I	T0/T1	N0	M0	188	1.97
	T0	N1	M0		
II	T2	N0	M0	560	5.86
	T0	N2a/N2b/N2c/N2NOS	M0		
III	T3	N0	M0	1784	18.67
	T1/T2/T3	N1	M0		
	T0	N3	M0		
IVA	T4a	N0/N1	M0	6049	63.31
	T1/T2/T3/T4a	N2a/N2b/N2c/N2NOS	M0		
IVB	*	N3	M0	581	6.08
	*	T4b	M0		
IVC	*	*	M1	392	4.10

Table S4 - 8th edition AJCC Staging Group after restaging with Supplemental Table 2

8th Stage Group	T Category	N Category	M Category	Frequency	Percent
**I**	**T0/T1/T2**	**N0/N1**	**M0**	1836	19.22
**II**	**T1/T1/T2**	**N2a/N2b/N2c/N2NOS**	**M0**	5326	55.75
	**T3**	**N0/N1/ N2a/N2b/N2c/N2NOS**	**M0**		
**III**	**T0/T1/T2/T3/T4a/T4b/T4NOS**	**N3**	**M0**	2000	20.93
	**T4a/T4b/T4NOS**	**N0/N1/ N2a/N2b/N2c/N2NOS/N3**	**M0**		
**IV**	*	*	**M1**	392	4.10
